# Construction and validation of a nomogram for predicting lateral lymph node metastasis in pediatric and adolescent with differentiated thyroid carcinoma

**DOI:** 10.1007/s12020-024-03730-6

**Published:** 2024-02-17

**Authors:** Jiaqiang Dan, Jingya Tan, Yao Guo, Yang Xu, Lin Zhou, Junhua Huang, Zhiying Yuan, Xiang Ai, Junyan Li

**Affiliations:** 1 Geriatric Diseases Institute of Chengdu/Cancer Prevention and Treatment Institute of Chengdu, Department of Thyroid and Breast Surgery, Chengdu Fifth People’s Hospital (The Second Clincal Medical College, Affiliated Fifth People’s Hospital of Chengdu University of Traditional Chinese Medicine), NO.33 Ma Shi Street, Wenjiang District, Chengdu, 611137 China; 2 Department of Rheumatology and Immunology, Wenjiang District People’s Hospital of Chengdu City, No.86, Kangtai Road, Wenjiang District, Chengdu, 611137 China; 3 Department of Thyroid and Breast Surgery, The General Hospital of Western Theater Command, No. 270, Day loop, Rongdu Avenue, Jinniu District, Chengdu, 610000 China

**Keywords:** Pediatric and adolescent, Differentiated thyroid carcinoma, SEER, Lymph node metastasis, Nomogram

## Abstract

**Background:**

Limited research has been conducted to specifically investigate the identification of risk factors and the development of prediction models for lateral lymph node metastasis (LNM) in pediatric and adolescent differentiated thyroid carcinoma (DTC) populations, despite its significant association with unfavorable prognosis.

**Methods:**

This study entails a retrospective analysis of the clinical characteristics exhibited by pediatric and adolescent patients who have been diagnosed with DTC. The data utilized for this analysis was sourced from the Surveillance, Epidemiology, and End Results (SEER) database, spanning the time frame from 2000 to 2020. Furthermore, the study incorporates patients who were treated at the Departments of Breast and Thyroid Surgery in the Second Clinical Medical College, Affiliated Fifth People’s Hospital of Chengdu University of Traditional Chinese Medicine, as well as The General Hospital of Western Theater Command, during the period from 2010 to 2020.

**Results:**

A cohort of 2631 patients from the SEER database, along with an additional 339 patients from our departments who met the specified inclusion criteria, were included in this study. Subsequently, four clinical variables, namely age, tumor size, multifocality, and extrathyroidal invasion, were identified as being significantly associated with lateral LNM in pediatric and adolescent DTC patients. These variables were then utilized to construct a nomogram, which demonstrated effective discrimination with a concordance index (C-index) of 0.731. Furthermore, the performance of this model was validated through both internal and external assessments, yielding C-index values of 0.721 and 0.712, respectively. Afterward, a decision curve analysis was conducted to assess the viability of this nomogram in predicting lymph node metastasis.

**Conclusion:**

The current investigation has effectively constructed a nomogram model utilizing visualized multipopulationsal data. Our findings demonstrate a significant association between various clinical characteristics and lateral LNM in pediatric and adolescent DTC patients. These outcomes hold substantial significance for healthcare practitioners, as they can employ this model to inform individualized clinical judgments for the pediatric and adolescent cohorts.

## Introduction

Pediatric and adolescent thyroid cancer is a rare form of cancer found in individuals under the age of 18 years, accounting for approximately 1.5% of all cancer cases in this age group [1]. The occurrence of thyroid cancer increases with age, although it is extremely uncommon before the age of 15 (0.4 per 100,000). However, it is now the most frequently diagnosed cancer in adolescents aged 15–19 (3.5 per 100,000) [2, 3]. Typically, pediatric and adolescent DTC presents as a symptomless lump in the thyroid, often discovered by their parents or the patients themselves. Consequently, DTC in this age group exhibits distinct clinicopathological characteristics when compared to DTC in adults. These unique features include extrathyroidal extension (ETE), bilateral involvement, multifocal lesions, and higher rates of lymph node metastasis(LNM) [4–8]. For pediatric and adolescent PTC cases with clinically negative lymph nodes, the occurrence of central LNM can be as high as 75% [9], and with reported prevalence rates reaching up to 83.3% [10]. Additionally, the rate of lateral LNM for pediatric PTC ranges from 34.2% to 62.5% [10–12]. According to various studies, in contrast to central LNM, lateral LNM was associated with recurrence of disease [13, 14].

Currently, based on the preoperative clinical characteristics and ultrasound (US) features, previous studies have identified several predictive factors for lateral LNM in pediatric and adolescent DTC patients. Notably, age, multifocality, tumor size, and extrathyroidal invasion showed significant associations with cervical lateral LNM [10–12]. However, it is crucial to acknowledge that the majority of these studies were conducted at a single center and had relatively small sample sizes, which may limit the generalizability and practicality of the model.

The objective of this study is to construct a nomogram model using extensive clinical characteristics obtained from the Surveillance, Epidemiology, and End Results (SEER) database, with the purpose of predicting the presence of lateral LNM in pediatric and adolescent DTC patients. Additionally, we created both an internal cohort and an external validation cohort within our departments to assess the predictive efficacy of this novel nomogram. The utilization of this comprehensive nomogram has the potential to aid clinicians in making informed decisions regarding treatment strategies, ultimately leading to improved patient outcomes.

## Materials and methods

### Data sources

The analysis conducted in this study relied on data obtained from two databases. Specifically, demographic characteristics and cancer incidence data pertaining to the American populations were extracted from the SEER program, which draws information from 18 cancer registries across the United States of America. These registries collectively cover approximately 28% of incident cases nationwide. This online program collects patient information and provides comprehensive details on diagnosis, demographics, tumor characteristics, initial treatment, and follow-up for vital status. Secondly, the external validation data were obtained from the Department of Breast and Thyroid Surgery’s electronic medical record system in the Second Clinical Medical College, Affiliated Fifth People’s Hospital of Chengdu University of Traditional Chinese Medicine and The General Hospital of Western Theater Command. Given that the SEER database is publicly accessible and the data used in this study was retrospectively analyzed from our electronic medical record system, informed patient consent was not required. Ethical approval was waived by the Ethics Committee of Second Clinical Medical College, Affiliated Fifth People’s Hospital of Chengdu University of Traditional Chinese Medicine and The General Hospital of Western Theater Command in view of the retrospective nature of the study.

### Patient selection

A screening program was conducted in SEER between 2000 and 2020 for patients with DTC. The variables that were examined in this study included the age at diagnosis, gender, race, tumor size, diagnostic confirmation, extrathyroidal invasion, laterality, multifocality, TNM stage (derived AJCC Stage Group, 7th edition), regional nodes examined, and regional nodes positive. The exclusion criteria consisted of patients who were older than 18 years, had other primary cancers, lacked histological confirmation results, did not undergo thyroidectomy, did not have regional lymph nodes examined, or had an unknown lymph node metastasis status. Additionally, patients with unknown race, tumor size, extrathyroidal invasion, and multifocality were also excluded.

External validation data sets were obtained from the multicenter cohort, which included the Second Clinical Medical College, Affiliated Fifth People’s Hospital of Chengdu University of Traditional Chinese Medicine, and The General Hospital of Western Theater Command (2010–2020). All the participants were Chinese. The inclusion criteria were the same as those of the SEER database.

### Evaluation of variables

Ultimately, 2163 patients from the SEER program and 339 patients from our institution were enrolled in this study after excluding unqualified patients. We screened the following variables from SEER for risk factors of cervical lateral LNM in DTC patients: age, race, gender, extrathyroidal invasion, multifocality, and tumor size. According to previous studies [6, 10, 12], the pediatric and adolescent populations were divided into age ≤15 years and >15 years groups. According to the degree and extent of tumor invasion, extrathyroidal invasion further subdivided T3b, T4a, and T4b, respectively. According to different size record, tumor size was further subdivided into ≤1 cm, 1–2 cm, 2–3 cm, 3–4 cm, 4–5 cm and >5 cm, respectively. In thyroid glands, multifocality means the presence of two or more sites, whereas a solitary tumor contains only one site.

### Statistical analysis

Both univariate and multivariate logistic regression models were employed to identify the factors associated with lateral LNM. Subsequently, a nomogram was developed using the statistically significant factors identified in the logistic regression model to predict the likelihood of lateral LNM for each patient. The predictive performance of the model was assessed through discrimination and calibration. The primary methods used to evaluate the level of discrimination and correction were the area under the curve (AUC) of the receiver operating characteristic curve (ROC curve) and the calibration curve. Using a decision curve analysis (DCA), the net benefit at different thresholds was calculated to determine the clinical utility of the nomogram.

For the statistical analyses, we employed IBM SPSS Statistics 24.0 (IBM Corporation, Armonk, NY, USA) and R version 4.3.0 software (The R Foundation for Statistical Computing, Austria, Vienna). A P value less than 0.05 was considered to have statistical significance.

## Results

### Baseline demographic and clinicopathological characteristics

A total of 2631 pediatric and adolescents patients (2121 female; 510 male) between 2000 and 2020 in the SEER program and 339 patients (265 female; 74 male) between 2010 and 2020 in the medical record of Second Affiliated Hospital Second Clinical Medical College, Affiliated Fifth People’s Hospital of Chengdu University of Traditional Chinese Medicine and The General Hospital of Western Theater Command were enrolled in this retrospective study. In order to develop and validate the nomogram, the data was divided into three distinct groups: the training set (consisting of 1273 diagnoses between the years 2000 and 2010), the internal validation set (consisting of 1358 diagnoses between the years 2011 and 2020), and the external validation set (consisting of 339 diagnoses between the years 2010 and 2020 from our two departments). The demographic and clinicopathological characteristics of these patients in the training and validation datasets at baseline were summarized in Table 1.

**Table 1:** Clinicopathological characteristics of pediatric and adolescent patients with DTC in SEER program and our centers

Variables	Subgroup	No. (%) of patients		
		Training data (*n* = 1273)	Internal testing (*n* = 1358)	External testing (*n* = 339)
Gender	Male	236 (18.5)	274 (20.2)	74 (21.8)
	Female	1037 (81.5)	1084 (79.8)	265 (78.2)
Race	White	1097 (86.2)	1114 (82.0)	/
	Black	34 (2.7)	54 (4.0)	/
	Other	142 (11.1)	190 (14.0)	/
				339 (100.0)
Age (year)	≤15	416 (32.7)	481 (35.4)	118 (34.8)
	>15	857 (67.3)	877 (64.6)	221 (65.2)
Tumor size (cm)	≤1	259 (20.3)	262 (19.3)	64 (18.9)
	>1 and ≤2	353 (27.7)	420 (30.9)	106 (31.3)
	>2 and ≤3	243 (19.1)	298 (21.9)	79 (23.3)
	>3 and ≤4	164 (12.9)	159 (11.7)	36 (10.6)
	>4 and ≤5	146 (11.5)	107 (7.9)	22 (6.5)
	>5	108 (8.5)	112 (8.3)	32 (9.44)
Extrathyroidal invasion	None	890 (69.9)	1088 (80.1)	303 (89.4)
	T3b	308 (24.2)	227 (16.7)	19 (5.6)
	T4a	39 (3.1)	38 (2.8)	15 (4.4)
	T4b	36 (2.8)	5 (0.4)	2 (0.6)
Lesions	Solitary	741 (58.2)	789 (58.1)	187 (55.2)
	Multifocal	532 (41.8)	569 (41.9)	152 (44.8)
Cervical lymph nodes	N0	451 (35.4)	474 (34.9)	115 (33.9)
	N1a	473 (37.2)	527 (38.8)	129 (38.1)
	N1b	349 (27.4)	357 (26.3)	95 (28.0)

### Predictors of lateral LNM

Finally, in the training group, approximately 27.4% (349/1273) of pediatric and adolescent patients were identified as presenting with lateral LNM, while the internal validation group had a rate of 26.3% (357/1358) and the external validation group had a rate of 28.0% (95/339) (Table 1). Univariate analysis revealed that age (*p* = 0.001), tumor size (*p* < 0.001), extrathyroidal invasion (*p* < 0.001), and multifocality (*p* < 0.001) were potential risk factors associated with lateral LNM in pediatric and adolescent DTC patients (Table 2). Subsequently, multivariate logistic regression was conducted to identify significant factors correlated with lateral LNM. Similarly, all of the variables we initially selected were consistent to be significantly correlated with lateral LNM (Table 2).

**Table 2:** Univariate and multivariate analysis of risk factors associated with lateral LNM

Variables	Subgroup	Univariate analysis		Multivariate analysis	
		Odds ratio	*p*	Odds ratio	*p*
Gender	Male	1			
	Female	0.895 (0.655–1.223)	0.487		
Race	White	1			
	Black	1.476 (0.721–3.020)	0.286		
	Other	1.099 (0.746–1.617)	0.634		
Age (year)	≤15	1		1	
	>15	0.658 (0.509–0.850)	0.001	0.727 (0.550–0.961)	0.025
Tumor size (cm)	≤1	1		1	
	>1 and ≤2	1.363 (0.904–2.056)	0.140	0.974 (0.632–1.502)	0.905
	>2 and ≤3	1.784 (1.159–2.746)	0.008	1.158 (0.730–1.836)	0.534
	>3 and ≤4	1.848 (1.153–2.962)	0.011	1.599 (0.976–2.618)	0.062
	>4 and ≤5	3.314 (2.085–5.265)	<0.001	1.307 (0.767–2.231)	0.327
	>5	5.884 (3.573–9.688)	<0.001	2.398 (1.349–4.263)	0.003
Extrathyroidal invasion	None	1		1	
	T3b	4.121 (3.108–5.465)	<0.001	3.269 (2.355–4.537)	<0.001
	T4a	6.660 (3.439–12.896)	<0.001	4.475 (2.214–9.042)	<0.001
	T4b	8.197 (4.064–16.531)	<0.001	4.652 (2.156–10.038)	<0.001
Lesions	Solitary	1		1	
	Multifocal	2.316 (1.803–2.976)	<0.001	1.984 (1.511–2.604)	<0.001

### Development and validation of the nomogram for lateral LNM

**Fig. 1:**
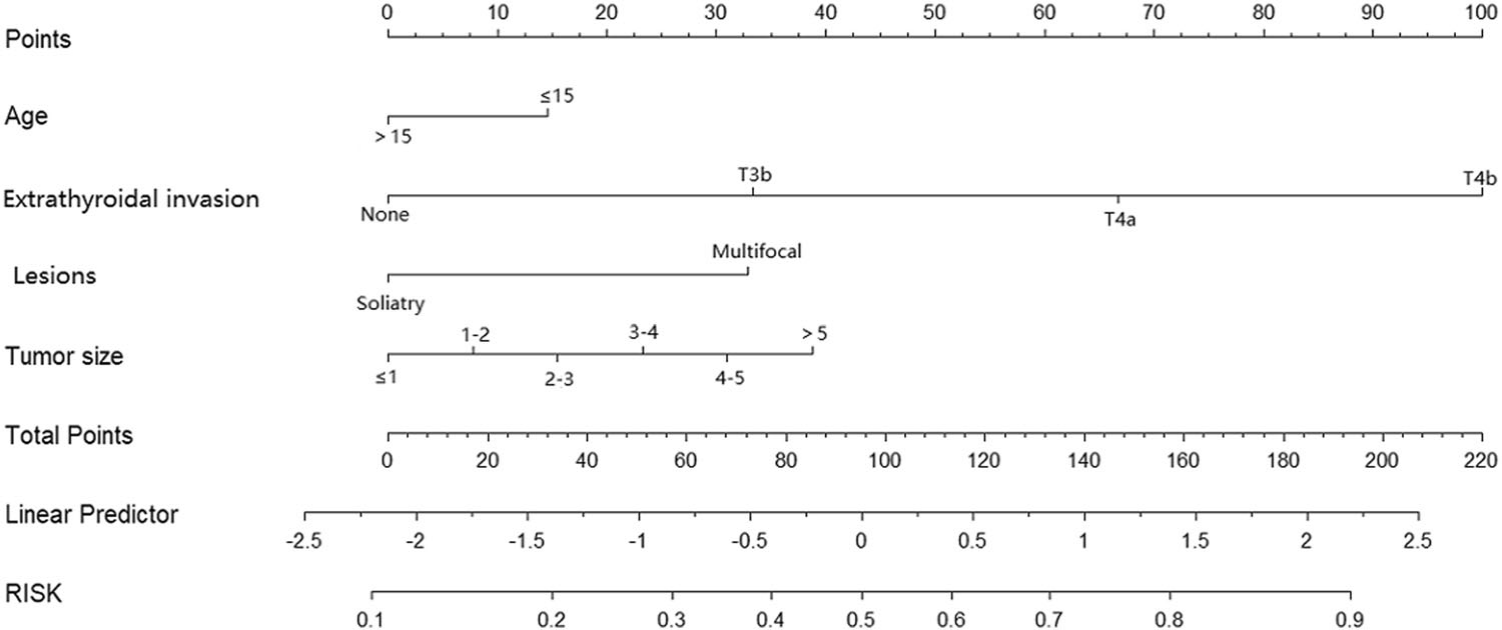
Nomogram to predict lateral LNM in pediatric and adolescent patients with DTC

Figure 1 illustrated the creation of a nomogram for multivariable logistic regression, incorporating factors such as age, tumor size, extrathyroidal invasion, and multifocality. To calculate the probability of lateral LNM, one can draw a vertical line and sum up the totals for each variable. In the nomogram, individuals aged 15 years or younger are assigned 15 points, whereas those older than 15 years receive 0 points. A multifocal lesion is assigned 32.5 points, while a solitary lesion receives 0 points. Tumor size less than or equal to 1.0 cm is assigned 0 points, whereas tumor sizes between 1 cm and 2 cm, 2 cm and 3 cm, 3 cm and 4 cm, 4 cm and 5 cm, and greater than or equal to 5 cm are assigned 7.5 points, 15 points, 22.5 points, 30 points, and 37.5 points, respectively. The absence of extrathyroidal invasion is assigned 0 points, while T3b, T4a, and T4b receive 34 points, 65 points, and 100 points, respectively. The total points axis can reach up to a maximum of 220, and the prediction capability of laternal LNM ranges from about 0.10 to 0.90.

### Internal and external validation of the nomogram

To validate the newly established nomogram for preoperative prediction of lateral LNM in pediatric and adolescent with DTC, additional validation was needed. To accomplish this, we constructed an internal validation cohort consisting of 1358 cases from the SEER program spanning the years 2011 to 2020, as well as an external validation cohort comprising 339 cases from our two departments between 2010 and 2020.

**Fig. 2:**
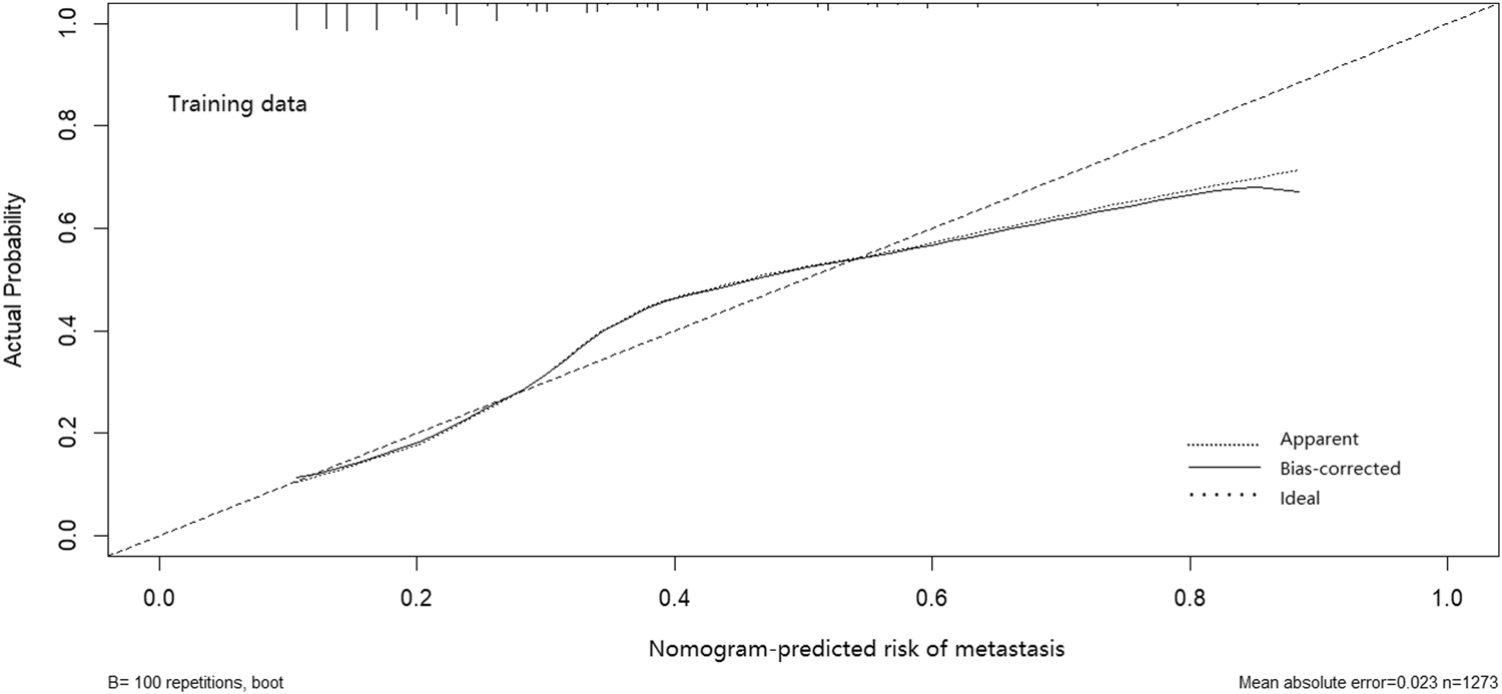
The calibration plot for probability of the lateral LNM nomogram

**Fig. 3:**
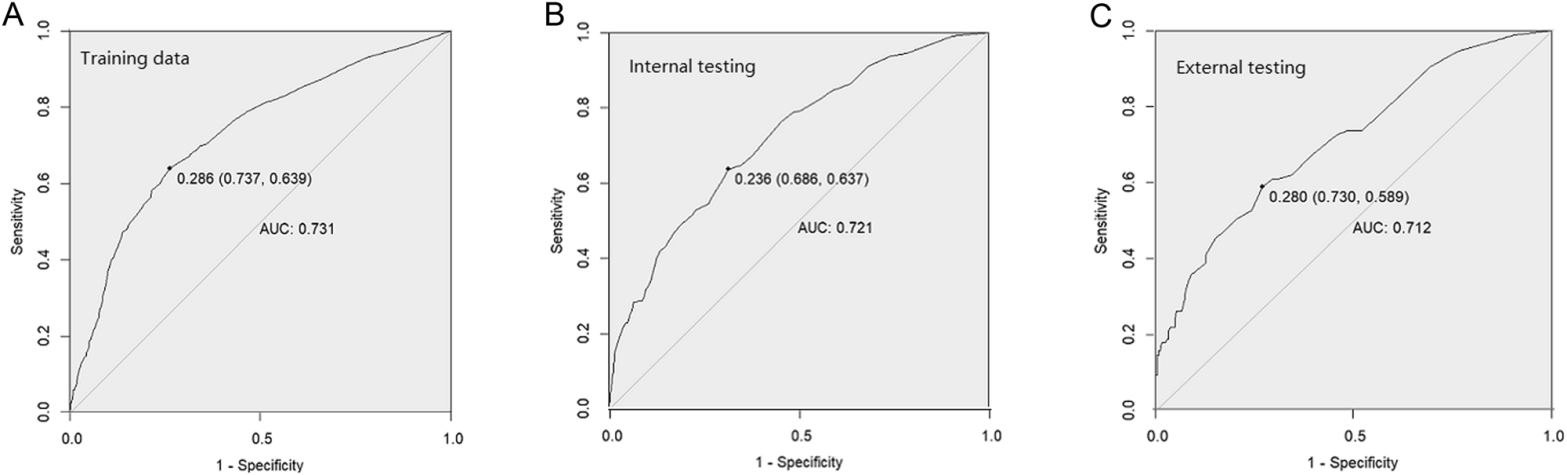
The receiver-operating characteristics (ROC) curve and area under the ROC (AUC) in the training cohort (**A**), internal cohort (**B**), and external cohort (**C**)

The calibration curve of the lateral LNM risk nomogram in the pediatric and adolescent DTC demonstrated a high level of agreement (Fig. 2). The C-index for the prediction nomogram was found to be 0.731, which was subsequently confirmed by 0.721 and 0.712 through internal and external validation, respectively, confirming its satisfactory performance of the nomogram in predicting lateral LNM. The receiver operating characteristics (ROC) curve and area under the ROC curve (AUC) were presented in Fig. 3A–C.

**Fig. 4:**
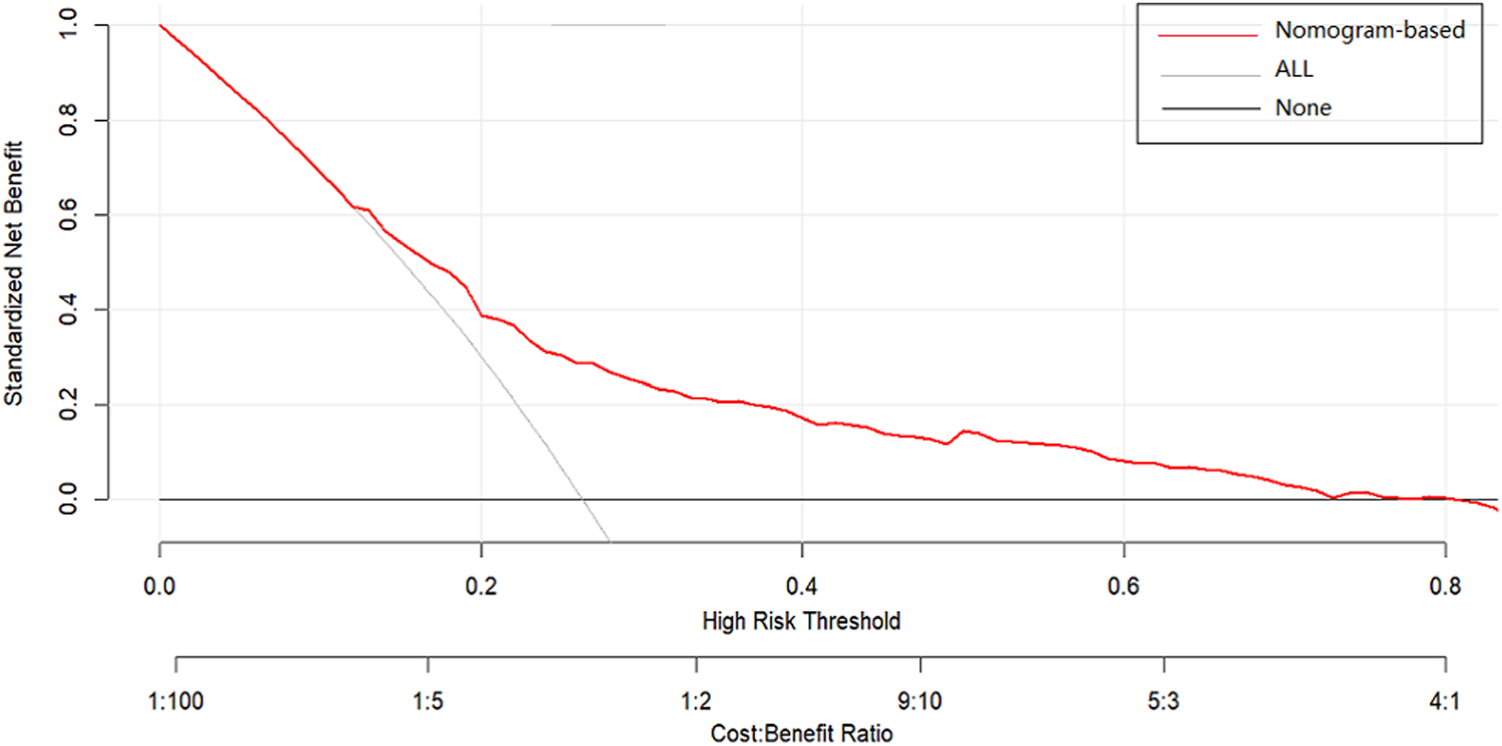
Decision curve analysis (DCA) for the risk nomogram. DCA shows that the model is clinically useful when intervention is decided in the threshold range of 10% to 75%

Furthermore, the clinical utility of the nomogram was assessed using DCA based on net benefit and threshold probabilities. The findings from DCA indicated that the utilization of this risk nomogram for predicting lateral LNM would be advantageous when the threshold probability falls within the range of 10% to 75% (Fig. 4). Within this range, the net benefit, as determined by the risk nomogram, demonstrated comparable values with overlapping results.

## Discussion

In this population-based study, we have identified four clinical characteristics that are significantly associated with an elevated risk of lateral LNM in pediatric and adolescent DTC patients. These characteristics included younger age, larger tumor size, extrathyroidal invasion, and multifocal disease. Leveraging these findings, we have successfully developed and validated the first nomogram for predicting lateral LNM in pediatric and adolescent DTC patients. This nomogram holds promise in assisting surgeons in making more informed intraoperative decisions regarding surgical options, especially concerning the necessity of intraoperative exploration or prophylactic lateral lymph node dissection (LND) in pediatric and adolescent DTC with cN0 or cN1a status.

The identification of these risk factors can aid in the identification of pediatric and adolescent patients with DTC who have a higher likelihood of having metastatic lateral lymph nodes. It is recommended that all patients diagnosed with DTC undergo thorough sonographic examination of the lateral cervical lymph node compartments to potentially detect clinical metastases. Extrathyroidal tumor extension should be specifically evaluated on preoperative sonography. Additionally, computed tomography (CT) also plays a significant role in determining the appropriate surgical approach, particularly for patients with lateral cervical LNM [15]. Patients without radiographic or clinical evidence of lymph node metastases should be thoroughly evaluated during surgery. Factors such as younger age, extensive extrathyroidal invasion, large tumor size, and known multifocal disease should be considered when deciding whether to perform intraoperative exploration or prophylactic lateral LND, since prophylactic lateral LND is not recommended for patients with cN0 or cN1a according to the 2015 guidelines from the American Thyroid Association (ATA) [6] and the 2022 European Thyroid Association Guidelines [16].

Previous studies have consistently shown a significant association was observed between tumor size and lateral LNM in pediatric and adolescent DTC. These studies have demonstrated that tumor size greater than 1 cm is a risk factor for lateral LNM, concurring with our results [10, 12, 14]. Moreover, our research further categorized tumor size into six subgroups: ≤1 cm, 1–2 cm, 2–3 cm, 3–4 cm, 4–5 cm, and >5 cm, respectively. Our study revealed that as tumor size exceeded 1 cm, the odds ratio (OR) for lateral LNM continued to rise, indicating a progressive increase in the incidence of lateral LNM with increasing tumor size. The implementation of a multi-stratification approach based on tumor size has the potential to yield more accurate assessments of the probability of lateral LNM. Notably, preoperative determination of tumor size through ultrasound facilitates a closer examination of the risk of lateral LNM for medical practitioners.

Some scholars also established a correlation between extrathyroidal invasion and probability of lateral LNM in both pediatric and adult DTC patients [10, 11, 17–19]. This current study highlights extrathyroidal invasion as the most significant risk factor for lateral LNM in pediatric and adolescent patients. Unlike previous investigations, our study further categorizes extrathyroidal invasion into T3b, T4a, and T4b based on T stage, which can be identified through preoperative ultrasound (US) and computed tomography (CT) scans, or intraoperative exploration. Similarly, the odds ratio for lateral LNM increases progressively in conjunction with the degree and extent of tumor invasion. Employing a multi-stratification method incorporating extrathyroidal invasion could offer more precise estimations of the likelihood of lateral LNM, a factor that has not been previously documented in existing literature.

Multifocal disease is more frequently found in pediatric and adolescent DTC patients than adults [7], and previous smaller-scale studies have consistently identified multifocal disease as a risk factor for lateral LNM in pediatric and adolescent patients [10, 12]. In this comprehensive population-level study, we further substantiated these findings by demonstrating that multifocal lesions carry a 2.316-fold higher risk of lateral LNM compared to solitary lesions.

Ngo et al. found that age ≤15 was initially identified as a potential risk factor for metastasis to lateral LNM in pediatric and adolescent DTC patients in their univariate analysis (*p* = 0.021). However, this association did not hold in their multivariate analysis (*p* = 0.468) [10]. It is possible that the relatively small sample size (*n* = 48) in their study compared to ours may have influenced these findings. In another investigation (*n* = 102) examining risk factors and prediction models for lateral LNM in pediatric and adolescent patients with papillary thyroid carcinoma, age was categorized into two groups: ≤15 years and 16–21 years. This division did not align with the age criterion for pediatric and adolescent patients (≤18 years) and finally did not demonstrate a significant association with lateral LNM [12].

Different from the above, it was demonstrated that age ≤15 years served as a noteworthy independent predictor for lateral LNM in our study. Also, in a recent investigation utilizing the SEER database to establish a correlation between age and the incidence of LNM in papillary thyroid cancer, especially in patients aged <30 years, Shukla et al. observed that younger age was linked to a higher number of positive nodes, an elevated lymph node ratio, and an increased risk of lateral neck disease [20]. These results are consistent with our own findings. Hence, our study’s extensive sample size enhances the persuasiveness of our findings, which indicate that younger age is an independent predictor for lateral LNM in pediatric and adolescent DTC patients.

The underlying mechanisms behind the higher rates of lateral LNM in younger patients have not been fully elucidated. Miccoli et al.’s findings indicate that younger age is associated with larger tumor size and a higher risk of thyroid capsule invasion, suggesting that tumors in younger patients exhibit more aggressive local behavior [21]. Vriens et al. proposed a molecular explanation for the variations in disease extent between younger and older patients, identifying several potential candidate genes that were differentially expressed in younger patients, notably showing lower expression of ECM1 [22], an extracellular matrix protein that may play a role in the metastatic pathway [23, 24]. Furthermore, certain studies have indicated that a younger age is associated with a greater likelihood of multiple genetic aberrations, suggesting that either younger patients are more vulnerable to multiple genetic events or multiple genetic events promote earlier disease onset and progression [25, 26].

Although several aforementioned studies have demonstrated a connection between tumor size, extrathyroidal invasion, multifocality and lateral LNM in pediatric and adolescent DTC populations, there is a scarcity of research that has examined data at the population level. Hence, we developed and validated a novel prognostic nomogram utilizing the four independent risk predictors to enable personalized prediction of lateral LNM. In order to establish a practical and applicable predictive nomogram model, a training cohort comprising 1273 patients from the SEER database was utilized. To further assess the applicability of our newly developed nomogram, we established both internal and external validation cohorts. Despite variations in the demographic characteristics of the study populations (American vs. Chinese-Han), the model exhibited satisfactory discrimination with a C-index of 0.712 in the external validation cohort, indicating its excellent performance.

Recently, Min et al. used the SEER database to establish nomogram for predicting the regional lymph node metastasis in the adolescent population [27]. Similar to our study, they found tumor size, extrathyroidal invasion and mltifocality were the independent factors for predicting the regional LNM in adolescents with DTC. However, regional LNM in this study encompassed both central and lateral lymph nodes, thereby diminishing the clinical significance of lateral LNM in pediatric and adolescent DTC, and their definition of adolescent age (between 10 and 24 years) did not meet the criteria of pediatric and adolescent. Additionally, the absence of external validation cohorts in their study further limits its reliability. Also, in another SEER database research to identify risk factors for nodal metastasis in pediatric DTC, Kim et al. found that increasing tumor size, extrathyroidal extension, and multifocal disease are independent factors associated with nodal metastases in pediatric DTC [28]. Similarly, nodal metastasis was not subdivided into central LNM and lateral LNM. What’s more, no nomogram was further developed.

Therefore, there were some noteworthy aspects warranting discussion: to the best of our knowledge, this was the first study to construct a nomogram model utilizing the SEER database, incorporating both internal and external validation cohorts, for the purpose of predicting lateral LNM of DTC in pediatric and adolescent populations. Since pediatric and adolescent DTC is uncommon compared to adult DTC, single-center studies have been limited by sample size. Conversely, our nomogram encompasses populations derived from the SEER database, encompassing multiple registered centers, diverse racial backgrounds, and substantial sample sizes, thereby enhancing the generalizability of this visualized model. Our study provides useful, population-level evidence to better risk stratify pediatric and adolescent DTC. Additionally, this nomogram was further validated by external cases from two centers.

Nevertheless, our study also has some limitations inherent to the SEER database. First, many patients were excluded from this study, because there were unknown and incomplete data. Second, information of ultrasound and CT features of laternal lymph nodes were not available in the SEER database. Finally, some laboratory testing variables such as carcinoembryonic antigen (CEA) and thyroglobulin antibody (TgAb) which have the potential to be associated with lateral LNM are not available. Furthermore, information of tumor’s specific location was not available in the SEER database. This is significant as DTC located in the upper pole is known to have a higher susceptibility to lateral LNM [17, 29, 30].

## Conclusions

To summarize, our study has successfully developed a predictive model that accurately classifies the likelihood of lateral LNM in pediatric and adolescent DTC patients. This model exhibits robust discriminatory and calibration abilities, making it a valuable tool for informing clinical decision-making in terms of personalized prognosis and treatment strategies.
